# Navigating the Complexity of PH-ILD: From Molecular Mechanisms to Integrated Clinical Evaluation

**DOI:** 10.3390/ijms27157055

**Published:** 2026-08-06

**Authors:** Eirini Vasarmidi, Diana Calaras, Ismini Kourouni, Apostolos Perelas, Katerina M. Antoniou

**Affiliations:** 1 Department of Respiratory Medicine, Laboratory of Molecular and Cellular Pneumonology, School of Medicine, University of Crete, 71500 Heraklion, Greece; 2 Department of Pulmonology and Allergology, State University of Medicine and Pharmacy “Nicolae Testemitanu”, MD-2004 Chisinau, Moldova; diana.calaras@usmf.md; 3 Division of Pulmonary, Critical Care and Sleep Medicine, Department of Medicine, MetroHealth Medical Center, Case Western Reserve University, Cleveland, OH 44109, USA; 4 Division of Pulmonary Disease and Critical Care Medicine, Virginia Commonwealth University, Richmond, VA 23298, USA

**Keywords:** interstitial lung diseases, pulmonary hypertension, group 3 PH, fibrotic lung diseases

## Abstract

Pulmonary Hypertension (PH) in patients with Interstitial Lung Disease (ILD) is a critical, yet underrecognized, complication that affects patients’ quality of life and increases mortality. Emerging evidence further suggests that PH-ILD is not merely a consequence of hypoxia and parenchymal fibrosis, as the severity of pulmonary vascular disease often correlates poorly with the extent of fibrotic lung involvement, indicating more complex underlying pathophysiological mechanisms. Diagnosis requires a high index of suspicion when symptoms appear “disproportionate” to the degree of parenchymal lung disease. Key indicators include diffusing capacity for carbon monoxide (DLCO) < 45%, a forced vital capacity to diffusing capacity for carbon monoxide ratio (FVC/DLCO) > 1.6, and radiological findings of increased pulmonary artery diameter. While echocardiography and circulating biomarkers serve as useful screening tools, right heart catheterization remains the gold standard for definitive diagnosis. Early identification is essential for risk stratification, lung transplant evaluation, and determining eligibility for targeted pharmacological interventions, as this group of patients remains one of the most therapeutically challenging forms of pulmonary vascular disease. Ongoing research and advances in diagnostic tools are increasingly focused on refining phenotypic classification, identifying valuable biomarkers, and elucidating molecular drivers that may enable personalized treatment strategies in this heterogeneous patient group.

## 1. Introduction

The 2022 ESC/ERS guidelines explicitly recommend further evaluation for pulmonary hypertension (PH) in patients with chronic lung disease, including interstitial lung disease (ILD), when symptoms, physiologic abnormalities, or imaging findings are disproportionate to the severity of parenchymal lung disease [1]. Notably, the coexistence of PH in chronic lung disease is often underrecognized, as the symptoms are nonspecific and commonly masked by underlying lung conditions and comorbidities, which tend to be more frequent in this population due to increased age [2], delaying diagnosis and intervention [3,4]. The coexistence of PH in patients with ILD dramatically worsens outcome. It is consistently identified as an independent predictor of mortality, with 3-year mortality rates of approximately 60–77%, highlighting the clinical importance of this complication [5,6]. A recent multicenter study found that male sex, functional class III or IV, pulmonary vascular resistance (PVR) > 5 Wood units, and absence of PH therapy were independent predictors of death or lung transplantation at 1 year in this group of patients [7].

Healthcare providers should maintain a high suspicion for PH when a patient presents with unexplained or progressive dyspnea, particularly if they also experience oxygen desaturation, reduced exercise capacity, or a slow heart-rate recovery following physical exertion. Further clinical clues include a disproportionately reduced diffusing capacity of the lungs for carbon monoxide (DLCO), increased biomarkers of cardiac stretch (brain natriuretic peptide (BNP) and/or NT-proBNP), and echocardiographic or radiographic features of PH [8,9]. Heightened clinical vigilance and a lower threshold for diagnostic evaluation are warranted in specific scenarios, particularly for patients exhibiting high-risk phenotypes or those experiencing acute clinical deterioration. Furthermore, a comprehensive assessment becomes imperative during the workup for lung transplantation or when evaluating a patient’s eligibility for PH-targeted pharmacological interventions and clinical trial enrollment.

PH in patients with ILD is frequently associated with advanced and extended fibrosis, although it can occur at any point during the disease course [10], reaching up to 86% in idiopathic pulmonary fibrosis (IPF) cohorts at the time of transplant evaluation [11]. High rates are reported in idiopathic nonspecific interstitial pneumonia (iNSIP) (31%) and chronic hypersensitivity pneumonitis (HP) (44%) [10,11], followed by connective tissue disease-associated ILD (CTD-ILD), sarcoidosis, pulmonary Langerhans cell histiocytosis (PLCH), and combined pulmonary fibrosis and emphysema (CPFE) [12,13,14]. Severe PH and a forced vital capacity (FVC) < 50% have been identified as independent predictors of survival, irrespective of the presence of emphysema in patients with CPFE [15]. Although CPFE patients exhibit more severe PH than those with IPF [16] or chronic obstructive pulmonary disease (COPD) alone [17], the prevalence of PH is not higher in CPFE than in patients with IPF matched for the extent of fibrotic lung disease [18]. On a larger scale, severe PH is considered relatively uncommon among patients with advanced ILDs, being found in less than 10% [1]. The discrepancies in reported PH prevalence among cohorts with ILD patients are primarily driven by heterogeneous diagnostic methodologies, fluctuating disease severity within study populations, and the evolution of PH hemodynamic definitions over time. Nevertheless, even mild to moderate PH carries substantial clinical significance in ILD, being associated with increased morbidity and mortality, as well as frequent hospitalizations [19].

Although the vast majority of ILDs are classified as group 3 PH, in systemic sclerosis (SSc), PH is typically categorized as group 1, although a variety of phenotypes may be present [20,21]. However, when SSc is associated with an ILD of increased severity, the related pathophysiologic mechanisms regard PH as group 3, and it has a significantly worse prognosis than SSc-PH without ILD [22]. Even though PH-ILD associated with CTD is not currently recognized as a distinct phenotype, its differentiation may be justified by its unique clinical characteristics and favorable prognosis compared with PH-ILD in the absence of autoimmune disease [23]. Meanwhile, sarcoidosis, lymphangioleiomyomatosis (LAM), and PLCH are classified under Group 5 [1]. This complex classification underlines the multifactorial nature and intricate interplay of mechanisms driving PH across the spectrum of ILD subtypes.

Current literature underscores a critical clinical requirement for refined, phenotype-specific diagnostic modalities to enhance identification and management across the ILD spectrum. Despite its high prevalence, PH in the context of ILD often remains underdiagnosed or undetected until the disease reaches advanced stages where therapeutic options are limited. The following section synthesizes the available evidence summarizing clinical, physiologic, and imaging markers as indicators of pulmonary vascular involvement in ILD, and their role in guiding risk stratification and diagnostic evaluation for PH.

## 2. Pathophysiological Background

### 2.1. Implicated Molecular Pathways

The mechanisms of PH in ILD remain incompletely understood. They are likely multifactorial, arising from the dynamic interplay between parenchymal injury, pulmonary vasculopathy, and the aggravating influence of coexisting comorbidities, driven by the synergistic action of dysregulated repair, chronic hypoxia, inflammation, and molecular abnormalities, ultimately converging on pulmonary vascular remodeling [3].

Within this complex pathobiological framework, increasing attention has focused on disrupted signaling pathways that normally maintain pulmonary vascular homeostasis. Among these, dysregulation of bone morphogenetic protein receptor type II (BMPR2)—a key component of the transforming growth factor β (TGF-β) superfamily—has emerged as a central molecular link between hypoxia, inflammation, aberrant repair, and the development of pulmonary vasculopathy. Although BMPR2 mutations are classically associated with heritable pulmonary arterial hypertension (PAH), an imbalance in BMPR-2/TGF-β signaling has been linked to the development of PH-ILD, highlighting the importance of acquired BMPR2 dysfunction [24].

In the pathogenesis of lung fibrosis, injured alveolar epithelial cells (AEC) promote the recruitment and activation of fibroblasts and myofibroblasts [25,26], which are major sources of TGF-β1 and excessive production of extracellular matrix (ECM) components. Persistent TGF-β1 signaling results in progressive tissue remodeling, and a vicious circle of endothelial dysfunction, smooth muscle cell proliferation, and ECM deposition within the vessel wall [27]. Excessive ECM creates a stiff microenvironment that promotes integrin-mediated activation of the TGF-β1 pathway, sustaining high levels of SMAD2/3 signaling [28]. Persistent TGF-β1 activity inhibits the BMPR2 activity, drives endothelial-to-mesenchymal transition (EndoMT), phenotypic switching in pulmonary artery smooth muscle cells (PASMC), and vascular fibrosis, contributing directly to increased PVR. Thus, reduced BMPR2 may contribute to both PH and fibrosis through excessive TGF-*β* signaling and this was also supported by a study showing that in patients with IPF, the BMPR2 levels were inversely correlated with mean pulmonary artery pressure (mPAP) [29].

Activins, also members of the TGF-β superfamily, particularly activin A, have emerged as key mediators linking parenchymal injury to pulmonary vascular disease. Elevated activin A levels have been observed in ILD and PH and are induced by inflammatory cytokines, hypoxia (via hypoxia-inducible factor-1α (HIF-1α), and mechanical stress within the fibrotic matrix [30]. In fibrotic ILD, activin A expression is increased in both serum and lung tissue and correlates with disease severity, driving fibroblast–myofibroblast transformation and pro-inflammatory signaling [31]. Moreover, dysregulated activin signaling activates SMAD2/3, functionally converging with TGF-β1 signaling, establishing a feed-forward loop in which inflammation, hypoxia, and fibrosis drive activin overproduction, which in turn suppresses BMPR2 and accelerates vascular disease progression. The recent approval of sotatercept, an activin signaling inhibitor, for the treatment of PAH further emphasizes the role of this pathway and the potential for use in PH-ILD [32].

Hypoxia arises from ECM accumulation that thickens the alveolar–capillary membrane and reduces the pulmonary capillary network, while fibrotic architectural distortion compresses adjacent vessels, collectively impairing oxygen diffusion. In hypoxic conditions, endothelial cells release endothelin-1 (ET-1) and decrease nitric oxide (NO) production, unbalancing the vascular tone. Moreover, chronic alveolar hypoxia induces cellular adaptation by stabilizing HIF-1α in pulmonary endothelial cells and PASMCs [33,34]. Activated HIF-1α promotes the expression of genes involved in inflammation, angiogenesis, vascular remodeling, and ECM synthesis, thereby contributing directly to pulmonary vascular remodeling and increased vascular resistance. Hypoxia suppresses BMPR2 signaling via transcriptional repression, epigenetic modifications, and BMPR2-targeting microRNAs, and enhances expression of SMAD2/3-activating ligands, including activins. Beyond biochemical signaling, increased ECM stiffness activates mechanotransduction pathways that promote smooth muscle proliferation and matrix synthesis while suppressing BMPR2-dependent growth restraint, even in the absence of hypoxia or inflammation [28]. Emerging evidence suggests that HIF-1α may serve as a potential treatment target for hypoxia-driven vasculopathy, while circulating levels of HIF-1α could have a role as a potential biomarker for early detection of PH-ILD, since serum concentrations have been shown to correlate with the extent of pulmonary microvascular injury [33,34].

Pro-inflammatory cytokines like tumor necrosis factor-a (TNF-α), interleukin-1β (IL-1β), interleukin-6 (IL-6), and interleukin-8 (IL-8) reduce BMPR2 expression in pulmonary vascular cells, mediated in part by NF-κB pathways, and drive vascular remodeling and PASMC proliferation in PAH models [35]. Particularly, IL-6 drives activation of the STAT3–microRNA-17/92 axis, which leads to post-transcriptional repression of BMPR2, with induction of miR-17-5p and miR-20a directly targeting BMPR2 mRNA, thereby reducing receptor expression and attenuating bone morphogenetic protein (BMP) signaling [36]. This cytokine-driven repression of BMPR2 shifts intracellular signaling toward SMAD2/3 (pro-proliferative) dominance. Inflammatory signaling via NF-κB and STAT3 further amplifies TGF-β1 and activin expression, reinforcing a pro-remodeling environment within the pulmonary vasculature [37]. While intact BMPR2 signaling is essential for maintaining pulmonary vascular homeostasis, this protective axis is highly vulnerable to disruption in the inflammatory milieu of fibrotic ILDs. In addition to hypoxia- and fibrosis-mediated mechanisms, chronic inflammation emerges as a potent upstream modifier of BMPR2 signaling. Importantly, experimental blockade of the IL-6/JAK/STAT3 axis reduced microRNA expression and attenuated fibrosis and vasculopathy, suggesting a promising therapeutic target for mitigating the progression of fibrosis-related diseases and vasculopathy [38].

### 2.2. Adaptive Hemodynamic and Structural Consequences of Vascular Remodeling

Thickening of the alveolar–capillary membrane and distortion of normal lung architecture impair oxygen diffusion and lead to loss and compression of the pulmonary capillary network. This reduction in the effective cross-sectional area of the pulmonary vascular bed increases resistance to blood flow and alters regional perfusion patterns. As fibrotic regions become poorly ventilated and less compliant, pulmonary blood flow is redistributed toward relatively preserved lung units, accompanied by abnormal neovascularization [39]. The newly formed vessels are often deficient in elastin and structurally irregular, further compromising vascular compliance [40]. This redistribution results in increased flow and shear stress within the remaining functional vessels, exposing the pulmonary endothelium to abnormal mechanical forces [41].

In early stages, hypoxic pulmonary vasoconstriction acts as a compensatory mechanism to optimize ventilation–perfusion matching; however, sustained hypoxia and chronic inflammation progressively transform this adaptive process into a maladaptive cascade with a persistent increase in pulmonary vascular tone [42]. This vascular remodeling at the structural and molecular levels can culminate in a PAH-like phenotype, in which the degree of hemodynamic impairment can be disproportionate to the extent of fibrosis [43].

**Figure 1:**
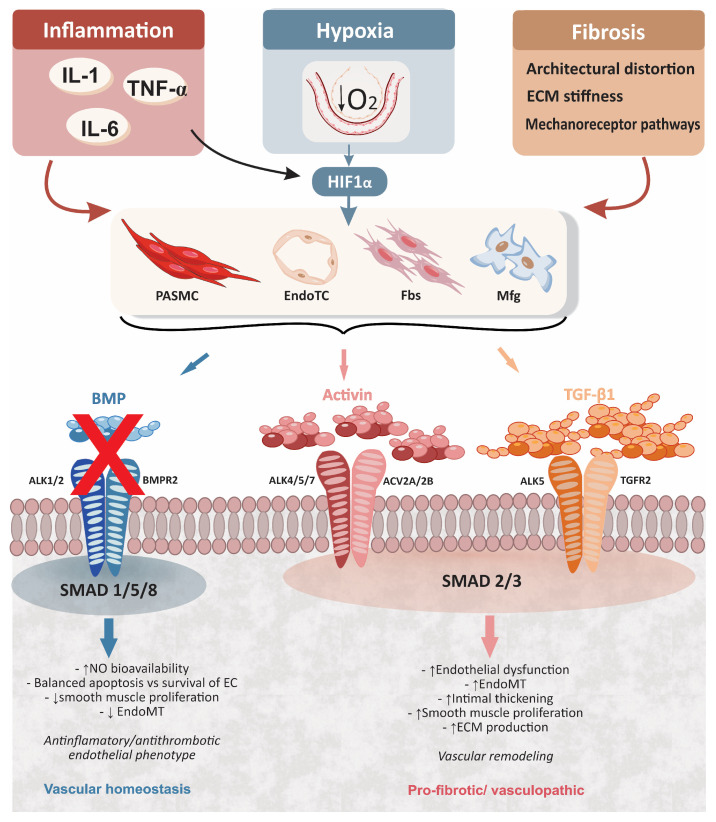
Convergent molecular pathways driving pulmonary vascular remodeling in pulmonary hypertension associated with interstitial lung disease (PH-ILD). Chronic inflammation, hypoxia, and fibrosis-related mechanical stress act synergistically to activate hypoxia-inducible and cytokine-driven signaling (notably via HIF-1α, IL-6, TNF-α), influencing multiple vascular cell types, including endothelial cells, pulmonary artery smooth muscle cells, fibroblasts, and macrophages. At the molecular level, these upstream stressors promote a shift in TGF-β superfamily signaling, characterized by suppression of the protective BMP–BMPR2–SMAD1/5/8 axis and dominance of pro-fibrotic activin/TGF-β–SMAD2/3 signaling. Loss of BMPR2-mediated signaling disrupts endothelial homeostasis, reduces nitric oxide bioavailability, and favors endothelial dysfunction and EndoMT, while enhanced SMAD2/3 signaling drives smooth muscle proliferation, intimal thickening, and excessive extracellular matrix deposition. Together, this imbalance establishes a pro-fibrotic and vasculopathic milieu, linking parenchymal lung injury to progressive pulmonary vascular disease and providing a mechanistic rationale for emerging therapies targeting BMPR2 restoration, activin/TGF-β modulation, hypoxia signaling, and extracellular matrix remodeling. Abbreviations: AEC—Alveolar epithelial cell, ALK—Activin receptor-like kinase, ACVR2A/2B—Activin A receptor type IIA/IIB, BMPR2—Bone morphogenetic protein receptor type II, BMP—Bone morphogenetic protein, EC—Endothelial cell, ECM—Extracellular matrix, EndoMT—Endothelial-to-mesenchymal transition, EndoTC—Endothelial cells (transitional/activated phenotype), Fbs—Fibroblasts, HIF-1α—Hypoxia-inducible factor 1 alpha, IL-1—Interleukin-1, IL-6—Interleukin-6, Mfg—Macrophages, NO—Nitric oxide, O_2_—Oxygen, PASMC—Pulmonary artery smooth muscle cell, SMAD1/5/8—Canonical BMP downstream signaling mediators, SMAD2/3—Canonical TGF-β/activin downstream signaling mediators, TGF-β1—Transforming growth factor beta-1, TGFBR2—Transforming growth factor beta receptor type II, TNF-α—Tumor necrosis factor alpha (Figure created using CorelDRAWSuite 2025).

Accumulating histopathological and translational evidence indicates that PH-ILD is not solely a downstream consequence of parenchymal fibrosis but rather reflects a diffuse pulmonary vascular disease that evolves in parallel with interstitial remodeling (Figure 1). The presence of multiple vascular phenotypes is plausible, as vascular abnormalities are not consistently observed within fibrotic regions and do not correlate with the severity of pulmonary fibrosis. These findings suggest that the pathogenic mechanisms underlying interstitial and vascular lung alterations are more intricate and interconnected than previously recognized [44]. Early histological observations demonstrated increased pulmonary arterial wall thickness in patients with PH-ILD not only in fibrotic regions but also in non-fibrotic lung areas when compared with ILD patients without PH [3]. Recent data reinforce this concept, showing that pulmonary vascular changes are widespread and involve endothelial dysfunction, medial hypertrophy, and adventitial thickening throughout the lung parenchyma, including relatively preserved areas [45]. This potentially explains the clinical observation that the severity of PH often correlates poorly with the extent of fibrosis on high-resolution computed tomography (HRCT) or with pulmonary function testing [46].

Apart from hypoxia, experimental and human studies propose additional mechanisms, including persistent endothelial injury, inflammatory and immune-mediated pathways, oxidative stress, impaired nitric oxide signaling, and dysregulated angiogenesis, all of which may promote progressive pulmonary vascular remodeling independently of parenchymal destruction [45]. These processes partially overlap with molecular and cellular pathways described in PAH, supporting the concept of shared pathobiological mechanisms between PH-ILD and PAH. Overall, distinguishing progressive vascular rarefaction as a consequence of advancing pulmonary fibrosis from pulmonary vascular remodeling that develops independently of fibrosis remains a fundamental pathogenic question that has yet to be fully elucidated.

## 3. Pulmonary Function Tests

Although no single non-invasive test establishes the diagnosis of PH-ILD, pulmonary function testing can provide an important clue. Given the presence of fibrosis, the most frequent abnormality observed is the reduction in forced vital capacity (FVC) and DLCO. Decreased DLCO though, indicates impaired gas exchange resulting from both alveolar-capillary fibrosis and/or pulmonary perfusion heterogeneity [47]. The role of pulmonary function testing in detecting PH-ILD has been an area of intense research, even though data mostly from retrospective studies remain somewhat conflicting. While a severe reduction in DLCO percent (%) (<45% predicted) can serve as an indicator for the presence of concomitant vascular disease, its performance in isolation has not always been optimal [48,49]. An FVC/DLCO ratio of >1.6 has been shown to predict the presence of PH in some series, as seen in SSc [50]. Moreover, the FVC/DLCO ratio has been shown to have a predictive value among patients with HP [51]. The derivative parameter obtained by adjusting DLCO for alveolar volume (KCO), in the absence of emphysema, demonstrates greater discriminative power for identifying pulmonary vasculopathy and a KCO ≤50% and/or a ≥15% decline over six months is strongly associated with a high probability of pulmonary hypertension [52,53].

Importantly, DLCO has emerged as the only pulmonary function parameter independently associated with survival; a multivariate analysis showed that patients with PH-ILD and DLCO < 32% predicted had 1- and 5-year survival rates of 68% and 13%, respectively [54]. However, it should be taken into consideration that changes in DLCO are non-specific and could be affected by coexisting conditions like anemia, cigarette smoking before the investigation, or chronic pulmonary embolism or emphysema [3]. Consequently, a careful and integrated interpretation of functional parameters is essential, and a disproportionately low DLCO relative to the extent of functional or radiological impairment in ILD should raise suspicion of concomitant PH [44,55].

## 4. Exercise-Based Assessment

Exercise testing provides important diagnostic and prognostic information in PH-ILD. The six-minute walk test (6MWT) is a safe, reproducible, and widely validated measure of functional exercise capacity in both clinical practice and research settings. Although it lacks diagnostic specificity for pulmonary vascular disease, reduced six-minute walk distance, exertional oxygen desaturation [56], and impaired heart rate—defined as a decrease of <13 beats per minute at 1 min post-exercise of the maximal—have been consistently associated with the presence of PH in IPF and with worse survival [57]. The minimal clinically important difference has been estimated to range from 24 to 45 m [58]. Accordingly, the 6MWT is best suited for assessing disease severity, longitudinal change, treatment response, and prognosis rather than for diagnostic discrimination [59].

Given that the 6MWT does not delineate the underlying mechanisms of exercise limitation, cardiopulmonary exercise testing (CPET) allows more precise characterization of pulmonary vascular dysfunction during exertion in PH-ILD. Exercise-induced PH, defined by a mPAP-to-cardiac-output slope > 3 mmHg·L^−1^·min^−1^, is observed in approximately 25–30% of patients with ILD and reflects increased PVR, reduced pulmonary compliance, and heightened right ventricular workload. CPET features associated with an increased likelihood of pulmonary vascular disease include reduced peak oxygen uptake ($\dot{V}$O_2_), an elevated ventilator equivalent for carbon dioxide ($\dot{V}$E/$\dot{V}$CO_2_) at the anaerobic threshold, and a low or flattened oxygen pulse ($\dot{V}$O_2_/heart rate) trajectory at peak exercise [60,61,62]. Collectively, these abnormalities support early detection of PH, refine disease severity, inform treatment response, and contribute to prognostic stratification, assuming the patient has sufficient fitness to perform [59].

In line with ESC/ERS group 3 PH recommendations, CPET complements standard testing by identifying pulmonary vascular limitation when exercise intolerance is disproportionate to parenchymal lung disease, thereby informing risk stratification and the need for invasive hemodynamic assessment [1,59]. In patients with ILD, invasive CPET (iCPET) enables direct characterization of pulmonary vascular reserve features that may remain occult at rest or on non-invasive testing. By measuring dynamic changes in pulmonary artery pressure, cardiac output, and PVR, iCPET allows definitive differentiation between precapillary, postcapillary dysfunction, and ventilatory or deconditioning-related causes of exercise intolerance [63,64]. In the context of suspected Group 3 PH, iCPET is particularly valuable when symptoms or functional limitation appear disproportionate to the degree of parenchymal lung disease and when resting hemodynamics are borderline or inconclusive and Group I or left heart contribution needs to be excluded [65,66].

**Figure 2:**
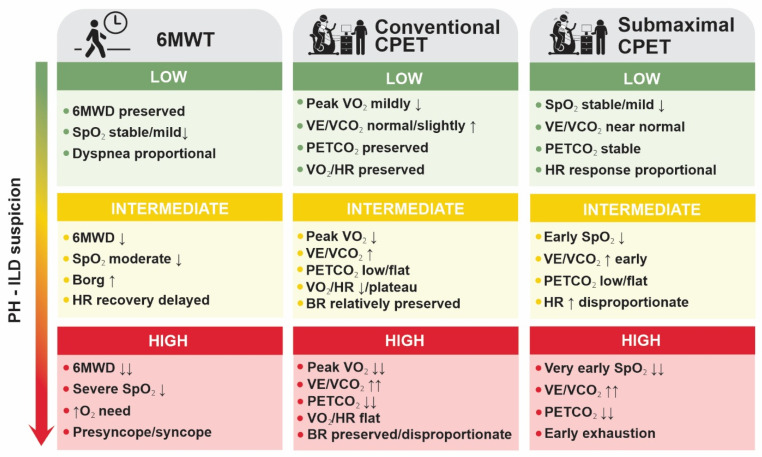
Multimodal exercise testing in pulmonary hypertension associated with interstitial lung disease (PH-ILD): A Comparison of six-minute walk test (6MWT) and cardiopulmonary exercise testing (CPET) Modalities. Across the three exercise modalities, increasing suspicion of PH-ILD is characterized by progressive reduction in exercise capacity, worsening exertional desaturation, increased oxygen requirement, ventilatory inefficiency, reduced or flattened PETCO_2_, impaired VO_2_/HR response, and disproportionate symptoms. The 6MWT mainly reflects global functional limitation and oxygen desaturation, whereas conventional CPET provides a more integrated assessment of ventilatory, circulatory, and gas-exchange abnormalities. Submaximal CPET may detect early pulmonary vascular or gas-exchange impairment at lower workloads, particularly in patients unable to achieve maximal effort. This stratification should be interpreted as a screening framework rather than a diagnostic classification, since confirmation of pulmonary hypertension requires right-heart catheterization. Abbreviations: ↓—decreased, ↓↓—severely decreased, ↑—increased, ↑↑—severely increased, PH—Pulmonary hypertension, ILD—Interstitial lung disease, 6MWT—Six-minute walk test, 6MWD—Six-minute walk distance, CPET—Cardiopulmonary exercise testing, SpO_2_—Peripheral oxygen saturation, VO_2_—Oxygen uptake/oxygen consumption, Peak VO_2_—Maximum oxygen uptake at peak exercise, VE—Minute ventilation, VCO_2_—Carbon dioxide output, VE/VCO_2_—Ventilatory equivalent for CO_2_, PETCO_2_—End-tidal carbon dioxide pressure, VO_2_/HR—Oxygen pulse, HR—Heart rate, BR—Breathing reserve, Borg—Borg dyspnea scale, O_2_ need—Oxygen requirement (Figure created using CorelDRAW).

Despite its diagnostic value, the routine clinical use of conventional CPET is limited by feasibility constraints, patient tolerance, and performance, particularly for patients with advanced ILD, oxygen dependence, or musculoskeletal limitations [67]. Submaximal CPET offers a more practical alternative, as it does not require maximal effort while remaining sensitive to pulmonary vascular dysfunction. By revealing a ventilation-perfusion mismatch, exchange inefficiency and increased dead space, submaximal CPET can identify pulmonary vascular disease earlier in its course. A recent study by Joseph P et al. showed promising evidence that a gas-exchange-derived estimate of pulmonary vascular capacitance (GXcap) ≤ 416 mL·mmHg, and delta end-tidal carbon dioxide (ETCO_2_) provide the best discrimination for detecting PH-ILD [68]. The authors suggest their multivariate analysis demonstrated high diagnostic accuracy, supporting its role as an adjunctive tool to guide invasive testing such as right heart catheterization (RHC) within a multimodal diagnostic framework. The complementary roles, physiologic targets, and practical considerations of the 6MWT, non-invasive CPET, and invasive CPET in PH-ILD are summarized in Figure 2.

## 5. Echocardiography Assessment

Transthoracic echocardiography (TTE) remains the most widely used non-invasive screening tool for initial cardiac assessment; however, reliance on right ventricular systolic pressure (RSVP) alone is limited by technical challenges, e.g., poor acoustic windows and suboptimal Doppler signals, resulting in poor correlation with invasive hemodynamics [69,70]. Contemporary approaches emphasize a more comprehensive evaluation of right ventricular structure and function rather than relying solely on estimated pulmonary pressure. Integration of parameters such as right ventricular size, tricuspid annular plane systolic excursion (TAPSE), fractional area change, interventricular septal configuration, and right atrial dimensions improves diagnostic performance, particularly for identifying more advanced vascular disease [71].

Multivariable echocardiographic models incorporating RVSP along with indirect markers of PH, such as right atrial enlargement, septal flattening, and altered ventricular geometry, have demonstrated superior accuracy for detecting severe PH in patients with ILD [72]. Another promising index is the systolic pulmonary artery pressure (sPAP) to pulmonary artery acceleration time (PAAT) ratio, which has been correlated with exercise capacity and PVR, as PAAT is considered an indirect surrogate of PVR. However, further data are required to validate this observation [73].

Despite these advances, TTE performs best in the presence of established right ventricular remodeling and systolic dysfunction. This represents an important limitation, as clinically meaningful elevations in pulmonary artery pressure may occur before overt right ventricular changes are evident, thereby affecting prognosis. In this context, it has been demonstrated that dysfunction of the right ventricle (RV) may represent an early manifestation of IPF that becomes more pronounced during exertion, as detected by two-dimensional speckle-tracking echocardiography [74]. This subclinical RV dysfunction has been associated with reduced exercise capacity, worsening pulmonary hemodynamics, impaired gas exchange, and lower DLCO [74].

**Figure 3:**
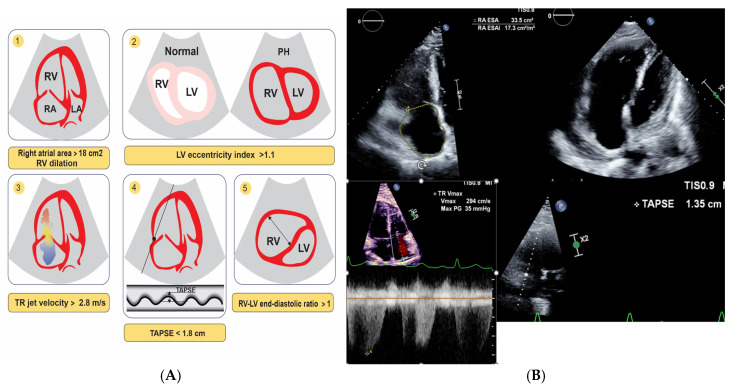
Transthoracic echocardiography signs of pulmonary hypertension associated with interstitial lung disease (PH-ILD). (**A**). The figure summarizes key echocardiographic parameters that increase suspicion of pulmonary hypertension by demonstrating right-sided chamber enlargement, right ventricular pressure overload, impaired right ventricular systolic function, and abnormal ventricular interaction. (1) Right atrial enlargement and right ventricular dilatation suggest chronic right-sided overload. (2) Septal flattening, expressed by an increased left ventricular eccentricity index indicates right ventricular pressure overload and altered interventricular geometry. (3) An increased tricuspid regurgitation jet velocity reflects an elevated pressure gradient across the tricuspid valve. (4) A reduced TAPSE suggests impaired right ventricular longitudinal systolic function, whereas (5) an increased RV–LV end-diastolic ratio reflects disproportionate right ventricular enlargement. Together, these findings provide a non-invasive echocardiographic framework for identifying patients with interstitial lung disease who require further evaluation for PH. (Figure created using CorelDRAW). (**B**). Representative image from a 70-year-old patient with idiopathic pulmonary fibrosis and PH-ILD showing the dilated right atrium (RA), the dilated RV (right ventricle) and the reduced tricuspid annular plane systolic excursion (TAPSE), along with an increased max velocity of the tricuspid regurgitation (TR) signal. Abbreviations: PH—Pulmonary hypertension, EchoCG—Echocardiography, RV—Right ventricle, RA—Right atrium, LV—Left ventricle, LA—Left atrium, TR—Tricuspid regurgitation, TR jet velocity—Tricuspid regurgitant jet velocity, TAPSE—Tricuspid annular plane systolic excursion, RV–LV ratio—Right ventricular to left ventricular diameter ratio, LV-eccentricity index—Left ventricular eccentricity index.

The limitations of echocardiography are underscored by studies comparing TTE with RHC in ILD populations, which have shown that a substantial proportion of patients classified as low probability for PH by ESC/ERS echocardiographic criteria nonetheless have hemodynamically confirmed disease [69]. These findings highlight the risk of false reassurance from a normal echocardiogram and reinforce the need for cautious interpretation. In everyday clinical practice, TTE should be considered a screening and risk-stratification tool with findings integrated with clinical features, pulmonary function testing, imaging, exercise physiology, and, when suspicion persists, invasive hemodynamic evaluation. The echocardiographic views and quantitative measurements mainly used in this integrated assessment are illustrated in Figure 3.

## 6. Circulating Biomarkers

Established circulating biomarkers, particularly BNP and N-terminal pro-BNP provide complementary information for risk stratification in PH-ILD. In accordance with ESC/ERS guidelines, these biomarkers should be viewed as indicators of cardiac stress and RV involvement rather than a diagnostic test [1]. Elevated BNP or NT-proBNP levels have been shown to predict mortality in mixed ILD cohorts and are associated with more advanced pulmonary vascular disease [75]. In patients with ILD, a BNP threshold >50 pg/mL has demonstrated moderate diagnostic performance for PH. In comparison, low NT-proBNP levels (<95 pg/mL) have a high negative predictive value, making clinically significant PH unlikely [76].

However, both biomarkers lack sensitivity for early disease and are frequently confounded by left-sided heart disease, left ventricular hypertrophy, acute pulmonary embolism, and acute coronary syndrome. Accordingly, BNP and NT-proBNP should be interpreted within a multimodal framework that integrates clinical features, pulmonary function testing, imaging, exercise capacity, and, when indicated, invasive hemodynamic assessment, rather than as a standalone screening or diagnostic tool.

## 7. Imaging Techniques and Composite Clinical Risk Scores

Complementing echocardiographic assessment of right ventricular structure and function, chest computed tomography (CT) provides additional anatomic markers that inform pulmonary vascular risk and disease proportionality in PH-ILD. Being routinely obtained in patients under workup for chronic lung disease and their longitudinal assessment (especially in ILD), imaging helps assess the proportionality between parenchymal lung disease and pulmonary vascular involvement, a key consideration for distinguishing Group 3 PH from Group 1 and Group 4, particularly in patients with CTD who are at risk for both. Increased attention has focused on leveraging CT-derived markers to identify patients at elevated risk who may benefit from invasive hemodynamic evaluation.

**Figure 4:**
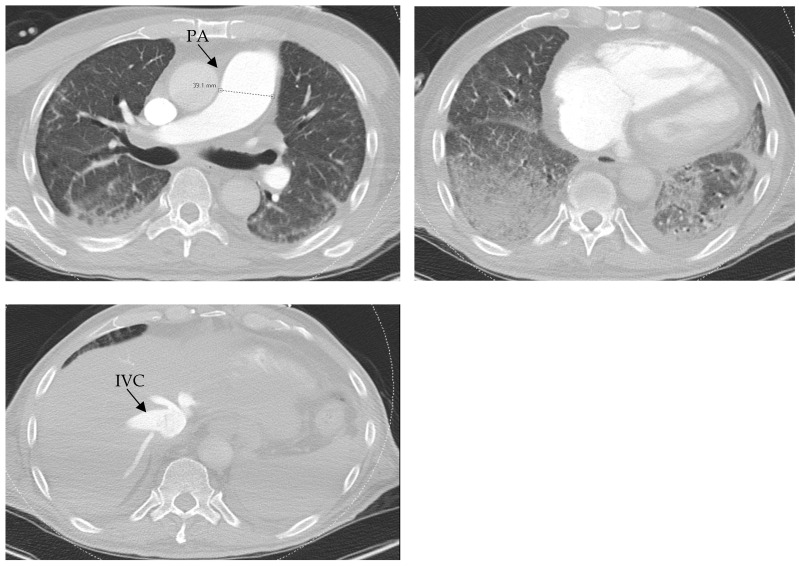
Signs in chest computed tomography (CT) of the lungs associated with pulmonary hypertension (PH). Representative images from a 58-year-old patient with Sjogren’s syndrome complicated by pulmonary hypertension associated with interstitial lung disease (PH-ILD). Upper panel—left: increased diameter of the pulmonary artery (PA, 39 mm), right: right atrial dilation and increased right ventricle to left ventricle diameter. Lower panel: reflux contrast into the inferior vena cava (IVC).

Among CT-derived metrics, enlargement of the main pulmonary artery (PA) quantified by absolute diameter or the pulmonary artery to ascending aorta (PA/AO) ratio, has been consistently associated with PH across the chronic lung disease population. Automated analysis of the PA volume has been feasible using specialized software is superior to PA/AO ratio [77]. As pulmonary vascular disease progresses, CT imaging captures adaptive and maladaptive cardiac changes, beginning with RV free wall hypertrophy (typically > 4 mm) and advancing to RV and atrial dilation, an increased right-to left ventricular diameter ratio, interventricular septal flattening or leftward bowing, and contrast reflux into the inferior vena cava and hepatic veins, reflecting elevated right sided filling pressures [78,79,80] (Figure 4).

Despite these associations, CT-derived PA metrics have important limitations. PA dimensions in ILD may be influenced by fibrotic traction, parenchymal distortion, hyperinflation, hypoxemia and acute clinical instability, reducing specificity for PH. As a result, the PA diameter can be elevated even in the absence of PH [7]. CT imaging obtained during clinical instability or flare may overestimate PH risk in the stable state [81,82]. Lastly, correlations between PA size and invasive hemodynamics are inconsistent, particularly in IPF and CTD-ILD, and weaken with advanced restrictive physiology (FVC ≤ 70% predicted) [82].

The clinical relevance of CT findings is strongly influenced by the pretest probability of PH. In patients with low clinical suspicion, large PA diameters (≥34 mm) or markedly elevated PA/AO ratios (>1.1) have limited specificity and should be interpreted cautiously [78]. In contrast, among individuals with intermediate or high pretest probability, including those with unexplained dyspnea, impaired gas exchange, or pulmonary vascular risk factors, PA enlargement of ≥30–32 mm or PA/AO ratio ≥ 0.9–1.0 warrants further evaluation for PH. Table A1 outlines a practical probability-based approach to evaluating incidental PA enlargement on chest CT and guiding subsequent diagnostic steps as recommended by the Fleischner Society [78].

Although cardiac magnetic resonance (CMR) is established for risk stratification and longitudinal assessment in PAH, its role in PH-ILD is weakly established. CMR provides an accurate and reproducible assessment of right ventricular hypertrophy, dilation, and systolic dysfunction, and registry data suggest it can identify severe PH and outperform echocardiography in selected patients with suboptimal transthoracic windows [83]. However, routine use is limited by availability and cost [78].

Composite clinical risk scores may help identify patients with ILD who are at increased risk of developing PH, particularly when symptoms, physiologic impairment, or imaging abnormalities appear disproportionate to the extent of parenchymal lung disease. The Zisman model, which incorporates oximetry along with FVC and DLCO percent predicted was validated in cohorts with advanced ILD, undergoing lung transplant evaluation, and may therefore be less sensitive for detecting earlier or less severe forms [84]. The FORD score based on routinely available clinical variables including the FVC/DLCO ratio, oxygen saturation nadir during the 6MWT, race, and six-minute walk distance, demonstrated moderate discriminative performance in the derivation and validation cohorts [85]. More recently, the PH-ILD detection score, which integrates multiple weighted clinical, physiologic, laboratory, and imaging parameters, has shown high diagnostic performance for stratifying patients into low-, intermediate-, and high-risk categories [86].

Collectively, these tools may support risk enrichment and timely referral for invasive evaluation; however, they should not be used in isolation and do not replace right heart catheterization, which remains the gold standard. The key components, derivation cohorts, and diagnostic performance of composite clinical risk scores for PH-ILD are summarized in Table A2.

## 8. Right Heart Catheterization in Group 3 Pulmonary Hypertension

Definitive characterization of pulmonary vascular disease in ILD ultimately requires invasive hemodynamic assessment, with RHC remaining the reference standard for diagnosis, phenotyping, and therapeutic decision-making. In accordance with ESC/ERS recommendations, RHC is required to distinguish precapillary from postcapillary hypertension, define disease severity and guide management and should be particularly considered at the diagnostic time points of clinical deterioration, worsening gas exchange, or functional limitation that appears disproportionate to the extent of parenchymal lung disease, as well as during lung transplantation evaluation [53]. Recent studies have explored the role of diagnostic and serial invasive hemodynamic assessment in ILD [85], suggesting that earlier detection of borderline or evolving pulmonary vascular abnormalities may help mitigate adverse outcomes associated with untreated PH, even in patients who are minimally symptomatic and may benefit from timely therapeutic intervention [87,88].

Given the high prevalence of concomitant left heart disease in chronic lung disorders, careful assessment of cardiac filling pressures is essential. Group 3 PH is hemodynamically defined by a mPAP > 20 mmHg, pulmonary capillary wedge pressure ≤ 15 mmHg, and PVR ≥ 2 Wood units [1]. Severe PH in chronic lung disease has been defined as an mPAP ≥ 35 mmHg, or mPAP ≥ 25 mmHg with a reduced cardiac index (<2.0 L/min/m^2^), a phenotype associated with circulatory-limited exercise intolerance and worse outcomes in ILD.

**Figure 5:**
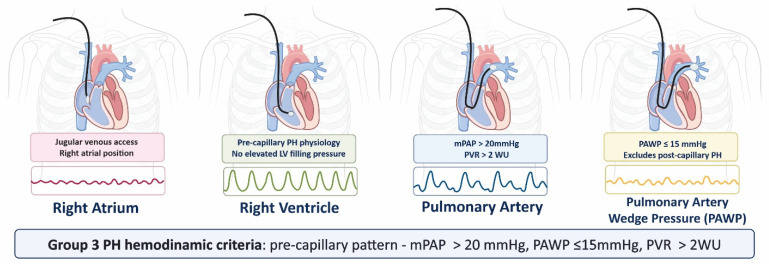
Hemodynamic assessment workflow during right heart catheterization. Abbreviations: PH—pulmonary hypertension, mPAP—mean pulmonary artery pressure, PCWP—pulmonary capillary wedge pressure, PH—pulmonary hypertension, LV—left ventricle, PVR—pulmonary vascular resistance, PAWP—pulmonary artery wedge pressure. (Figure created using CorelDRAW).

RHC should be performed when the underlying parenchymal lung disease is stable and is generally well-tolerated even among patients with advanced ILD, including those who require supplemental oxygen [89]. Pulmonary capillary wedge pressure should be measured over multiple respiratory cycles and recorded at end-expiration to account for the marked respiratory variation [90] (Figure 5). In patients with risk factors for postcapillary physiology—such as systemic hypertension, atrial fibrillation, obesity, obstructive sleep apnea, or echocardiographic evidence of diastolic dysfunction- provocative maneuvers, including fluid loading or exercise, may aid diagnostic clarification. Emerging data suggest that PVR may provide superior prognostic information compared with mPAP alone in patients with ILD [91]. Routine acute vasodilator testing is not recommended in Group 3, given the lack of therapeutic implications and potential risks [1]. Differentiating Group 1 PAH from Group 3 PH remains challenging, particularly in CTD, and requires a multidisciplinary and multimodal approach. Left heart catheterization and left ventricular end-diastolic pressure (LVEDP) measurement should be considered in cases where pulmonary veno-occlusive disease is suspected and it can be performed at the same time as the RHC.

**Figure 6:**
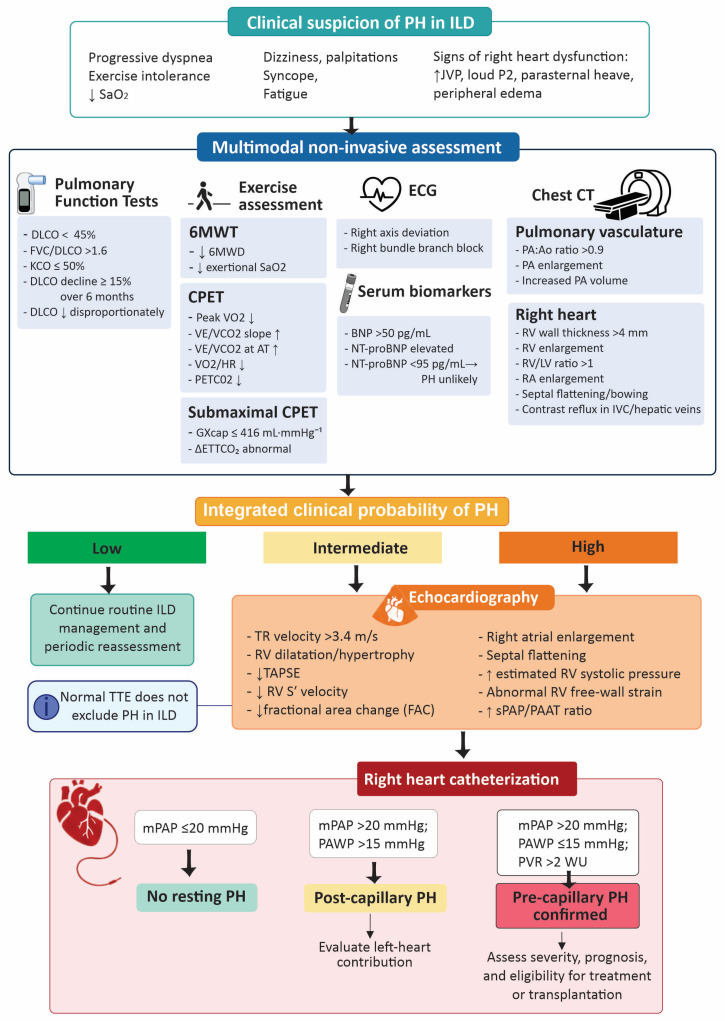
Proposed diagnostic algorithm for the detection of pulmonary hypertension (PH) in a patient with interstitial lung disease (ILD). From symptoms and signs increasing clinical suspicion to the final diagnosis based on the gold standard: right heart catheterization. Abbreviations: 6MWD—six-minute walk distance, 6MWT—six-minute walk test, AT—anaerobic threshold, BNP- B-type natriuretic peptide, CPET—cardiopulmonary exercise testing, CT—computed tomography, DLCO—diffusing capacity of the lungs for carbon monoxide, ECG—electrocardiography, ETCO_2_—end-tidal carbon dioxide, FAC—fractional area change, FVC—forced vital capacity, GXcap—gas-exchange-derived estimate of pulmonary vascular capacitance, ILD—interstitial lung disease, IVC—inferior vena cava, JVP—jugular venous pressure, KCO—carbon monoxide transfer coefficient, LV—left ventricle, mPAP—mean pulmonary artery pressure, NT-proBNP—N-terminal pro-B-type natriuretic peptide, P2—pulmonary component of the second heart sound, PA—pulmonary artery, PA/AO—pulmonary artery-to-ascending aorta diameter ratio, PAAT—pulmonary artery acceleration time, PAWP—pulmonary artery wedge pressure, PETCO_2_—end-tidal carbon dioxide pressure, PH—pulmonary hypertension, PVR—pulmonary vascular resistance, RA—right atrium, RHC—right heart catheterization, RV—right ventricle, RV S′—right ventricular peak systolic myocardial velocity, SaO_2_—arterial oxygen saturation, sPAP—systolic pulmonary artery pressure, TAPSE—tricuspid annular plane systolic excursion, TR—tricuspid regurgitation, $\dot{V}$E/$\dot{V}$CO_2_—ventilatory equivalent for carbon dioxide, $\dot{V}$O_2_—oxygen uptake, $\dot{V}$O_2_/HR—oxygen pulse, WU—Wood units. (Figure created using CorelDRAW).

Table 1 integrates functional, imaging, and invasive markers in the evaluation of PH-ILD, summarizing their diagnostic value and role in risk stratification across the diagnostic pathway. Figure 6 proposes an evidence-based diagnostic algorithm that incorporates the diagnostic tools described in the preceding sections.

## 9. Novel and Emerging Treatment Strategies

PH associated with chronic lung disease represents one of the most therapeutically challenging forms of PH. Unlike PAH, PH Group 3 develops in the context of heterogeneous parenchymal lung disease, where vascular remodeling, hypoxia, inflammation, and fibrosis coexist and interact. This complexity limits the applicability of PAH-targeted therapies and underlies the historically disappointing results observed in clinical trials. Therefore, the therapeutic landscape of PH-ILD is undergoing a conceptual transition from nonspecific repurposing of PAH therapies toward phenotype-oriented treatment strategies. Multiple randomized trials of systemic PAH therapies in ILD—including endothelin receptor antagonists and soluble guanylate cyclase stimulators—have yielded neutral or harmful results [43,92]. The early termination of RISE-IIP due to excess mortality with riociguat firmly established that systemic vasodilation in fibrotic lung disease can be deleterious, likely by exacerbating ventilation–perfusion mismatch [92]. These failures have been instrumental in shaping contemporary drug development paradigms, emphasizing regional selectivity, phenotype matching, and avoidance of indiscriminate vasodilation.

To date, inhaled treprostinil remains the only therapy with robust randomized controlled trial evidence demonstrating clinical benefit in PH-ILD. In the INCREASE trial, inhaled treprostinil significantly improved 6-min walk distance and reduced disease-progression events, supporting the principle that lung-selective vasodilation can improve functional capacity without worsening ventilation–perfusion mismatch [93]. In contrast, the parallel PERFECT trial in PH-COPD was terminated early due to harm, underscoring the heterogeneity of Group 3 PH and the importance of disease-specific pathophysiology [94]. The benefit from the inhaled treprostinil seemed to span across ILD diagnoses, perhaps except for CPFE that seemed to gain the least benefit from the intervention [95]. Although the treprostinil effect is more prominent among individuals with a higher PVR (>4 Wood units), it has been shown that even patients with less hemodynamic compromise can derive benefit [95].

Beyond the primary endpoint, post-hoc and extension analyses of INCREASE have yielded several insights relevant to future treatment strategies. First, composite disease-progression analyses suggested fewer ILD exacerbations and functional declines with inhaled treprostinil, suggesting that vascular modulation may indirectly influence parenchymal disease trajectories [93]. This was further supported by the recent positive results of the TETON-2 trial [95] in which inhaled treprostinil was associated with a reduction in FVC decline among patients with IPF, regardless of PH status. Second, signals of stabilization or modest improvement in FVC, particularly in IPF subgroups, support emerging concepts of vascular–fibrotic cross-talk, although these findings remain hypothesis-generating. However, certain limitations temper these observations. In the open-label extension, patients initially randomized to treprostinil maintained functional stability, whereas those switching from placebo did not show comparable improvement, suggesting that late initiation in advanced PH-ILD may have limited reversibility [93]. On the basis of these findings, the 2022 ESC/ERS guidelines recommend inhaled treprostinil in carefully selected patients with PH related to lung disease and hypoxia [1]. Moreover, the medication tolerance is less than optimal; cough is a particularly bothersome side effect and is a leading cause for medication discontinuation. Patients also have expressed concerns about effects on their daily activities and their quality of life; the medication is administered via a specialized nebulizer multiple times a day resulting in significant time commitment and need to plan activities around the dosing schedule. Although the development of a dry powder inhaler can improve adherence, it has been associated with increased frequency and severity of cough.

Thus, the success of inhaled treprostinil has redefined the vasodilator paradigm by emphasizing pulmonary-selective delivery. This has catalyzed the development of next-generation inhaled prostacyclin formulations, while reinforcing continued caution toward systemic vasodilators in unselected Group 3 PH populations. Second-generation formulations—treprostinil palmitil inhalation powder (TPIP) and liposomal treprostinil—aim to optimize delivery, duration of action, and adherence.

Targeting the NO–sGC–cGMP pathway represents a parallel vasodilatory strategy. Mosliciguat, an inhaled apo-sGC activator, is designed to restore cGMP signaling even under oxidative stress, where endogenous NO signaling is impaired [96]. Ongoing PH-ILD trials emphasize PVR reduction as a primary endpoint, signaling a more hemodynamically focused development strategy.

While pulmonary-selective vasodilation remains an important therapeutic pillar in PH-ILD, it does not directly address the underlying vascular remodeling that drives disease progression. Consequently, attention has shifted toward disease-modifying strategies that target the proliferative and inflammatory processes that sustain pulmonary vascular pathology. Seralutinib, a tyrosine kinase inhibitor (TKI), targets platelet-derived growth factor receptor (PDGFR), colony-stimulating factor 1 receptor (CSF1R), and c-KIT, pathways implicated in pulmonary vascular remodeling, macrophage activation, and fibroproliferative signaling [97]. The SERANATA trial, a randomized, placebo-controlled trial investigates seralutinib in a PH-ILD population. Similar to Treprostinil formulations, Seralutinib is delivered by inhalation, attempting to localize anti-remodeling effects to the pulmonary circulation while avoiding systemic toxicity.

Among emerging therapeutic concepts, restoration of BMP signaling through activin pathway modulation represents a paradigm shift. Sotatercept, an activin receptor type IIA (ActRIIA) fusion protein, acts as a ligand trap for activins and growth differentiation factors, rebalancing signaling away from pro-proliferative SMAD2/3 pathways toward BMP-mediated growth inhibition. In the phase 2 PULSAR trial and subsequent phase 3 studies in PAH, sotatercept produced marked reductions in pulmonary vascular resistance and improvements in functional and biomarker endpoints when added to background therapy [32]. Although these trials were conducted in Group 1 PAH, their relevance to PH-ILD lies in the increasing recognition of a vascular-dominant or PAH-like PH-ILD phenotype, characterized by disproportionate pulmonary vascular remodeling, BMPR2 dysfunction, and activin pathway activation. Preclinical and translational data demonstrating activin A upregulation in pulmonary vascular endothelium and fibrotic lung tissue provide biological plausibility for extending this approach to selected PH-ILD subgroups [30].

Preliminary observational data in sarcoidosis-associated pulmonary hypertension further suggest potential benefits of activin pathway modulation beyond classic PAH, although these findings remain exploratory and require prospective validation [98]. These findings position activin signaling as a future target for selected PH-ILD phenotypes, particularly those with disproportionate vascular disease.

Several emerging agents designed to modulate oxidative stress, mitochondrial dysfunction, inflammatory signaling, or extracellular matrix remodeling have translated these upstream biological insights into targeted therapeutic strategies currently under clinical investigation. Mirivadelgat, an aldehyde dehydrogenase 2 (ALDH2) activator, targets toxic aldehyde accumulation and mitochondrial injury—mechanisms implicated in both ILD progression and pulmonary vascular dysfunction [99]. Similarly, pioglitazone, a PPAR-γ agonist, is being explored in chronic lung disease-PH (CLD-PH) as a bioenergetic modulator [100,101]. Although primarily mechanistic, such studies reinforce the notion that metabolic reprogramming may influence the trajectory of pulmonary vascular disease.

Hymecromone, studied in the SATURN trial, demonstrates that it is safe and well-tolerated in patients with both Group 1 and Group 3 PH, with no treatment-related adverse events or discontinuations over 24 weeks. Although the primary hemodynamic endpoint (change in PVR) was not met, the study provides a clinically meaningful exploratory signal in Group 3 PH, where treated patients showed a substantial improvement in functional capacity, with an unadjusted mean increase in 6MWD of 66 m compared with deterioration in the placebo group, alongside consistent improvements in quality-of-life scores [102].

**Figure 7:**
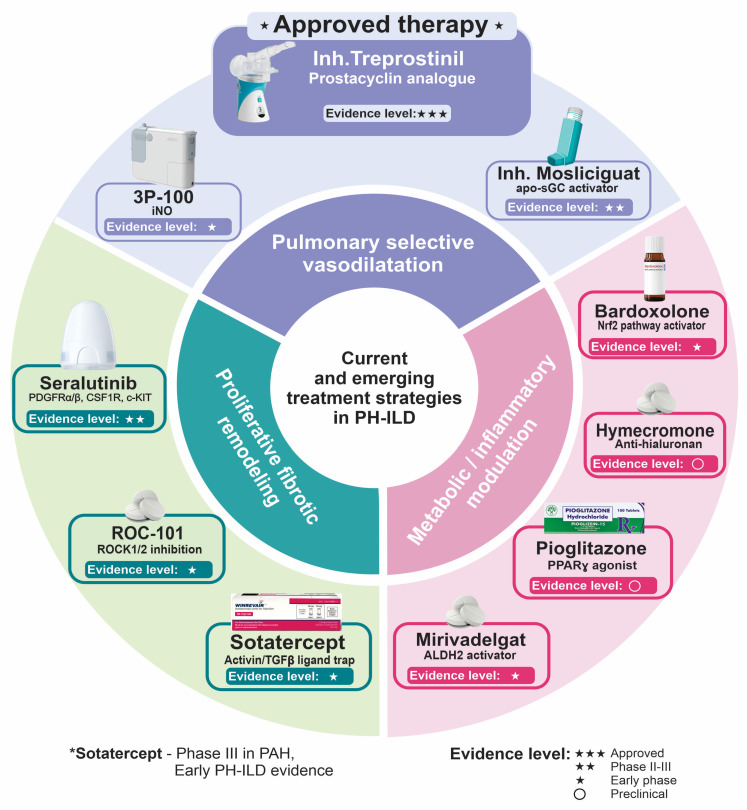
Current and emerging treatment strategies in pulmonary hypertension associated with interstitial lung disease (PH-ILD). Therapeutic domains that reflect dominant pathobiological mechanisms: Pulmonary-selective vasodilatation (blue sector)—targets impaired vasomotor tone and ventilation–perfusion mismatch through lung-restricted activation of the NO–sGC–cGMP and prostacyclin pathways. Representative agents include inhaled treprostinil (prostacyclin analogue), inhaled mosliciguat (apo-sGC activator), and inhaled nitric oxide delivered via the 3P-100 device, aiming to improve pulmonary hemodynamics while minimizing systemic hypotension. Proliferative/fibrotic remodeling (green sector) addresses structural pulmonary vascular remodeling, driven by smooth muscle proliferation, endothelial dysfunction, and dysregulated TGF-β superfamily signaling. Agents in this category include seralutinib (PDGFRα/β, CSF1R, c-KIT inhibition), ROC-101 (ROCK1/2 inhibition), and sotatercept (activin/TGF-β ligand trap), all designed to counteract maladaptive vascular and fibroproliferative pathways. Metabolic/inflammatory modulation (pink sector)—targets upstream metabolic stress, oxidative injury, and extracellular matrix dysregulation, which amplify both fibrosis and vasculopathy. Key examples include hymecromone (hyaluronan synthesis inhibition), mirivadelgat (ALDH2 activation), pioglitazone (PPARγ agonism), and bardoxolone methyl (Nrf2 pathway activation). Inhaled treprostinil is the only approved therapy for PH-ILD and is distinguished from investigational agents through a visual evidence-grading system (★★★ = approved therapy; ★★ = Phase II–III clinical development; ★ = early-phase clinical development; ○ = preclinical evidence). (Figure created using CorelDRAW).

Figure 7 and Table A3 provide an overview of emerging treatment modalities and active clinical trials.

## 10. Conclusions and Future Directions

The challenges in addressing PH in fibrotic ILD are multifaceted. The heterogeneity of ILDs, as well as the effect of PH on prognosis and patients’ quality of life, emphasize the significant unmet need for reliable, early biomarkers that can identify individuals at risk before overt clinical manifestations. Symptom overlap between PH and ILD makes early recognition difficult, while diagnostic tools such as echocardiography have limited accuracy in fibrotic lungs, and RHC remains invasive and not widely accessible.

Accumulating clinical evidence challenges the traditional paradigm of PH-ILD being viewed as a hypoxia-driven consequence of parenchymal destruction leading to secondary vascular remodeling [46]. The severity of PH often shows poor correlation with the extent of fibrotic lung disease, and some patients develop severe pulmonary vascular disease despite relatively preserved lung volumes and limited radiographic fibrosis [103]. Therapeutic strategies are equally challenging: antifibrotic agents slow lung function decline but do not target pulmonary vascular pathology, while recent trials with vasodilators are heterogeneous, with benefit observed in selected subgroups but neutral or even harmful effects in others [46,93]. These discrepancies strongly suggest that PH-ILD is not a monolithic condition but rather a spectrum of overlapping yet distinct disease phenotypes.

Integrating hemodynamic data, lung function patterns, imaging characteristics, gas-exchange abnormalities, and circulating biomarkers offers a pragmatic approach to refined phenotyping. Such a strategy aligns with broader trends in pulmonary vascular medicine toward precision, mechanism-based care and may improve prognostication, therapeutic selection, and clinical trial design.

Phenotyping PH-ILD has direct diagnostic and therapeutic relevance [44]. Patients with a vascular-dominant phenotype often exhibit disproportionate pulmonary vascular resistance, marked DLCO–FVC dissociation, and relatively limited fibrosis on HRCT, and may derive benefit from PH–targeted therapies, as demonstrated in the INCREASE trial of inhaled treprostinil [93]. Failure to recognize these distinctions likely contributes to the mixed outcomes of PH-ILD clinical trials, in which biologically dissimilar patients are analyzed as a single group. Current international guidelines acknowledge the complexity of PH-ILD but largely rely on severity-based definitions rather than mechanistic stratification.

Mechanistic studies indicate that PH-ILD comprises distinct phenotypes, including a vascular-dominant PAH-like form driven by intrinsic vasculopathy, acute and chronic pulmonary emboli, neovascularisation, and a parenchymal-dominant phenotype mediated by chronic alveolar hypoxia, hypoxic vasoconstriction, parenchymal architectural disruption, capillary loss, and V/Q mismatch [104]. Importantly, these mechanisms are not mutually exclusive. Their relative contribution varies across individuals, resulting in distinct hemodynamic profiles, lung function patterns, imaging features, and molecular signatures. Furthermore, the comorbidity profile represents an additional determinant of disease heterogeneity. Emerging biomarkers such as activin A, HIF-1α, interleukin-6, and BMPR2-regulating microRNAs further support the presence of biologically meaningful differences within the PH-ILD population that are not captured by hemodynamic thresholds alone [30,33,36].

Emerging diagnostic tools include advanced imaging and data-driven techniques beyond established echocardiographic and CT-based assessments. Novel imaging technologies are expanding the non-invasive evaluation of ILD and PH. Radiomics and artificial intelligence (AI) represent a transformative advance in thoracic imaging, enabling the extraction of high-dimensional quantitative features and automated pattern recognition. Radiomics-based models have demonstrated prognostic value in ILD, including CTD-associated cohorts [105]. Automated volumetric analysis of the PA has been shown to predict the presence of PH with reasonable accuracy. At the same time, AI-driven CT analysis improves the detection of complex imaging patterns that may be under-recognized by visual assessment [43]. AI-driven analysis of the electrocardiogram is currently being investigated as a non-invasive tool for the detection of PH (NCT06911632).

Dual-energy computed tomography (DECT) enables the generation of pulmonary iodine maps as non-invasive surrogates of regional perfusion. While most clinical applications have focused on chronic thromboembolic disease [42], quantitative assessment perfused blood volume may offer indirect markers of pulmonary vascular remodeling and altered hemodynamics. However, the utility of DECT-derived perfusion metrics in Group 3 PH remains incompletely studied and requires further validation. Hyperpolarized ^129^Xe MRI is another promising functional imaging modality that provides spatial and quantitative metrics of pulmonary ventilation, perfusion and hemodynamics. Although still in its infancy in PH, it has been helpful in disease detection and can be used to monitor response to therapy [106]. Together, these approaches hold promise for early identification of pulmonary vascular involvement and more individualized risk stratification, although prospective validation is needed before routine clinical implementation.

Future research should prioritize establishing multinational registries and conducting of studies aimed at early identification of PH, refined risk stratification, and the elucidation of the underlying pathophysiological and molecular mechanisms driving disease development and progression. Comprehensive deep phenotyping of PH-ILD subgroups and the utilization of novel diagnostic modalities is also warranted to improve disease characterization and facilitate precision medicine approaches. Advances in these areas are expected to inform the development of innovative clinical trial designs and clinically meaningful endpoints, ultimately accelerating the identification and implementation of novel therapeutic strategies for this high unmet medical need.

Taken together, PH in fibrotic ILD represents a major challenge in respiratory medicine. It is a condition that not only magnifies disease burden but also exposes gaps in timely diagnosis, risk stratification, and evidence-based management. Addressing this concern requires greater awareness, refined diagnostic algorithms, and effective, targeted treatment strategies to improve survival and quality of life for this vulnerable patient population.

## Figures and Tables

**Table 1:** Functional, imaging, and invasive markers for diagnostic evaluation in patients with pulmonary hypertension associated with interstitial lung disease (PH-ILD).

Domain	Parameter	Key Findings Suggestive of PH-ILD	Clinical Utility	Limitations
Clinical	Symptoms	Disproportionate dyspnea Fatigue Syncope Worsening exercise tolerance	Increased suspicion → screening	Non-specific Overlap with ILD progression
	Physical exam	JVD Peripheral edema Parasternal heave Loud P2	Indicates advanced disease	Late findings Poor sensitivity for early PH
Pulmonary Function	Spirometry/Lung volumes	No consistent correlation with PH presence	ILD severity assessment	Degree of restriction does not predict PH
	DLCO	DLCO < 40% predicted Rapid decline DLCO disproportionate to FVC; ↑ FVC/DLCO	Strong screening marker Prognostic marker	Influenced by emphysema and anemia
Exercise Testing	6-min walk distance (6MWD)	Reduced distance Worsening over time	Index of functional status Prognostic marker	Non-specific Effort dependent
	Oxygen desaturation (6MWT)	Marked exertional desaturation	Suggests vascular limitation	Also reflects ILD severity
	Heart rate recovery	<13 bpm at 1 min post-6MWT	Marker of PH Suggestive of adverse outcomes	Not routinely assessed
	CPET (invasive/non-invasive	Ventilatory inefficiency (↑ VE/VCO_2_) Increased dead space Reduced O_2_ pulse	Differentiates vascular vs parenchymal limitation	Limited availability Logistical complexity
	Submaximal CPET	Abnormal ventilatory efficiency Reduced GX-derived pulmonary vascular capacitance (GXCAP)	Promising screening adjunct	Limited validation Not widely available
HRCT	Fibrosis extent	Poor correlation with PH	ILD characterization	Cannot exclude PH
	Pulmonary artery diameter	PA/AO ratio > 0.9	Suggests pulmonary vascular disease	Affected by age and body size
	Cardiac chamber size	RV/LV ratio > 1 Septal flattening	Indicates RV pressure overload	Less sensitive for early disease
	Advanced CT models	PA size RVOT thickness Septal angle	Risk stratification (under research)	Limited ILD-specific validation
Echocardiography	TR velocity/RVSP	Elevated estimated RVSP	Screening tool	Often inaccurate or unobtainable in ILD
	RV size/function	↓ TAPSE ↑ RA area ↓ RV FAC	Markers of advanced PH	May be normal in early PH-ILD
	Composite echo scores	Multivariable RV-focused models	Improved detection of severe PH	Limited sensitivity for mild PH
Cardiac MRI	RV volumes and mass	↑ RV mass index Septal angle changes	Gold standard RV assessment	Cost Limited access Requires expertise
	PA flow dynamics	Reduced PA distensibility	Prognostic marker Clarifies phenotype	Limited clinical availability
Composite Scores	FORD score	FVC/DLCO O_2_ nadir Race 6MWD	Non-invasive risk stratification	Moderate accuracy
	PH-ILD detection tool	Weighted clinical functional echo parameters	High sensitivity High specificity	Requires validation in broader cohorts
Invasive Hemodynamics	RHC	mPAP elevation ↑ PVR normal PAWP (precapillary PH)	Diagnostic gold standard	Invasive Requires expertise
	Exercise RHC or fluid challenge	Unmasks occult postcapillary PH	Clarifies phenotype	Not universally performed

Abbreviations: PH—Pulmonary hypertension, ILD—Interstitial lung disease, JVD—Jugular vein distension, DLCO—Diffusing capacity of the lungs for carbon monoxide, FVC—Forced vital capacity, 6MWD—Six-minute walk distance, 6MWT—Six-minute walk test, CPET—Cardiopulmonary exercise testing, VE—Minute ventilation, VCO_2_—Carbon dioxide output, VE/VCO_2_—Ventilatory equivalent for CO_2_, GXCAP—Gas Exchange-derived pulmonary vascular capacitance, CT—Computed tomography, HRCT—High resolution computed tomography, PA/AO ratio—Pulmonary artery to ascending aorta diameter ratio, RV/LV ratio—Right ventricular to left ventricular diameter ratio, PA—Pulmonary artery, RVOT—Right ventricular outflow tract, TR—Tricuspid regurgitation, RVSP—Right ventricular systolic pressure, RV—Right ventricle, TAPSE—Tricuspid annular plane systolic excursion, RA—Right atrium, RV FAC—Right Ventricular Fractional Area Change, RHC—Right heart catheterization, mPAP—mean pulmonary artery pressure, PVR—Pulmonary vascular resistance, PAWP—Pulmonary artery wedge pressure.

## Data Availability

No new data were created or analyzed in this study. Data sharing is not applicable to this article.

## Appendix A

**Table A1:** Interpretation of Incidentally Identified Pulmonary Artery Enlargement on Chest Computed Tomography (CT) According to Pretest Probability of Pulmonary Hypertension (PH).

Clinical Context (Pretest Probability for PH)	Incidentally Observed Main Pulmonary Artery Diameter	Incidentally Observed PA/AO Ratio	Suggested Interpretation
Low Likelihood	≥34 mm	≥1.1	May be abnormal, but has low specificity for PH; interpret cautiously
Intermediate Likelihood	≥32 mm	≥1.0	Raises suspicion for PH; it should prompt correlation with symptoms, lung function, and echocardiography
High Likelihood	≥30 mm	≥0.9	Strongly suggestive of underlying PH in an appropriate clinical context
Established Clinical Suspicion of PH	Any value	Any value	PA size alone is insufficient; definitive diagnosis requires invasive hemodynamic assessment

Abbreviations: PH—Pulmonary hypertension, PA/AO ratio—Pulmonary artery to ascending aorta diameter ratio.

**Table A2:** Comparison of Non-Invasive Screening Scores for Detection of Pulmonary Hypertension in patients with Interstitial Lung Disease (PH-ILD).

Feature	Zisman Model	Ford Model	PH-ILD Detection Score
Primary Purpose	PH risk in advanced ILD populations	Estimate the likelihood of PH using functional parameters	Comprehensive screening Risk stratification for PH-ILD
Target Population	Advanced ILD Lung transplant candidates	ILD patients undergoing functional evaluation	Broad ILD population, including CTD-ILD
Number Of Variables	3	4	8
Key Domains Included	Pulmonary function Oxygenation	Pulmonary function Exercise capacity Oxygenation Demographics	Clinical symptoms Pulmonary function Exercise capacity Imaging Biomarkers
Pulmonary Function Variables	FVC (% predicted) DLCO (% predicted)	FVC/DLCO ratio	DLCO (% predicted) FVC (% predicted)
Exercise Variables	None	6-min walk distance	6-min walk distance
Oxygenation Variables	Resting pulse oximetry	Oxygen nadir during 6-min walk test	Supplemental oxygen use
Biomarkers	None	None	NT-proBNP
Imaging Variables	None	None	Pulmonary artery enlargement on CT
Clinical Features	None	Race	Syncope/presyncope
CTD-ILD Consideration	Not included	Not included	Included
Scoring Output	Continuous probability model	Continuous point-based score	Categorical score (low/intermediate/high risk)
Risk Thresholds	Probability-based	Score correlates with PH likelihood	≤3 low 4–5 intermediate ≥6 high risk
External Validation	Yes	Yes	Yes
Strengths	Simple Minimal variables Useful in advanced ILD	Uses readily available functional data	High sensitivity and specificity Multimodal
Limitations	Validated mainly at the transplant-level disease	Limited clinical and imaging integration	Slightly more complex Requires biomarker data
Best Clinical Role	Screening for advanced ILD Transplant evaluation	Early functional screening	Prioritization for right heart catheterization

Abbreviations: PH—Pulmonary hypertension, ILD—Interstitial lung disease, CTD—Connective tissue disease, FVC—Forced vital capacity, DLCO—Diffusing capacity of the lungs for carbon monoxide, NT-proBNP—N-terminal pro-B-type natriuretic peptide, CT—Computed tomography.

**Table A3:** Summary of ongoing clinical trials in the field of pulmonary hypertension associated with interstitial lung disease (PH-ILD).

Trial/NCT/Active Substance	Investigational Therapy	Phase	Mechanism of Action	Population/Phenotype	PH Confirmation	Endpoints
SAPPHIRE (NCT03814317)	Inhaled treprostinil	II	Prostacyclin analogue → pulmonary vasodilation, anti-proliferative effects	Sarcoidosis-associated PH (often ILD overlap)	RHC required	6MWD NT-proBNP QoL Safety
TPIP/PALM-ILD (NCT07179380)	Treprostinil palmitil inhalation powder	III	Long-acting prostacyclin prodrug, once-daily pulmonary delivery	PH-ILD	RHC required	Exercise capacity NT-proBNP Safety
SATURN (NCT05128929)	Hymecromone (H01)	IIa	Inhibits hyaluronan synthesis, targeting ECM accumulation, vascular stiffness, and fibro-vascular coupling	PH with and without ILD	RHC required	Change in PVR 6MWD Biomarkers Safety
Pioglitazone CLD-PH (NCT06336798)	Pioglitazone	II	PPAR-γ agonist → improves mitochondrial bioenergetics, anti-inflammatory and metabolic reprogramming	CLD-PH (incl. ILD-PH)	RHC required	Change in skeletal muscle and pulmonary bioenergetics Exercise capacity Biomarkers
SOTERIA (NCT04796337)	Sotatercept	III	Activin/TGF-β ligand trap → restores BMPR2–SMAD1/5/8 signaling; anti-proliferative	Sarcoidosis-associated PH	RHC confirmed	Safety 6MWD NT-proBNP Shift or maintenance in WHO Functional Class Change in PVR Survival
PHocus (NCT06635850)	Mosliciguat	II	Inhaled apo-sGC activator, NO-independent cGMP generation	PH-ILD	RHC required	Change in PVR 6MWD NT-proBNP
PHactor NCT07333183	Mosliciguat + inhaled Treprostinil (iTre)	II	Mosliciguat: inhaled apo-sGC activator restoring NO–sGC–cGMP signaling under oxidative stress; treprostinil: prostacyclin pathway agonism (vasodilation/antiproliferation)	PH-ILD on background iTre	RHC required	Safety Tolerability
WINDWARD (NCT06475781)	Mirivadelgat	II	ALDH2 activation → reduces toxic aldehydes, mitochondrial protection	PH-ILD	RHC required	Change in PVR (Week 12) 6MWD NT-proBNP
SERANATA (NCT07181382)	Seralutinib	III	Inhaled TKI (PDGFRα/β, CSF1R, c-KIT) → anti-proliferative, anti-fibrotic	PH-ILD	RHC required	Exercise capacity Hemodynamics Biomarkers
ROCSTAR (NCT07175038)	ROC-101	IIa	ROCK1/2 inhibition → anti-contractile, anti-fibrotic	PAH and ILD-PH	RHC required	Change in PVR 6MWD NT-proBNP WHO functional class

Abbreviations: 6MWD—6-min walk distance, ALDH2—aldehyde dehydrogenase 2, apo-sGC—heme-free soluble guanylate cyclase, cGMP—cyclic guanosine monophosphate, c-KIT—KIT proto-oncogene receptor tyrosine kinase (CD117), CLD-PH—pulmonary hypertension associated with chronic lung disease, CSF1R—colony-stimulating factor 1 receptor, ECM—extracellular matrix, ILD—interstitial lung disease, iNO—inhaled nitric oxide, iTre—inhaled Treprostinil, NO—nitric oxide , NT-proBNP—N-terminal pro-B-type natriuretic peptide, PAH—pulmonary arterial hypertension, PDGFRα/β—platelet-derived growth factor receptor alpha/beta, PH—pulmonary hypertension, PH-ILD—pulmonary hypertension associated with interstitial lung disease, PPAR-γ—peroxisome proliferator-activated receptor gamma, PVR—pulmonary vascular resistance, QoL—quality of life, RHC—right heart catheterization, ROCK1/2—Rho-associated coiled-coil-containing protein kinase 1/2, sGC—soluble guanylate cyclase, TKI—tyrosine kinase inhibitor, WHO—World Health Organization.
